# Depressive and anxiety symptoms during the COVID-19 pandemic in the oldest-old population and the role of psychosocial factors: a multivariate and multi-wave analysis

**DOI:** 10.3389/fpubh.2023.1229496

**Published:** 2023-12-15

**Authors:** Sina K. Gerhards, Alexander Pabst, Melanie Luppa, Steffi G. Riedel-Heller

**Affiliations:** Institute of Social Medicine, Occupational Health and Public Health (ISAP), University of Leipzig, Leipzig, Germany

**Keywords:** mental health, social support, old age, COVID-19, pandemic

## Abstract

**Background:**

Since the oldest-old population was identified as a high-risk group for a severe course of the coronavirus disease and higher mortality, it was assumed that they might be particularly psychologically burdened. The aim of the study is to analyze the development of anxiety and depressive symptoms over the course of the pandemic from 2020 to 2022, as well as psychosocial factors associated with these outcomes.

**Method:**

We analyzed data of *n* = 135 participants aged 78 to 97 years old (2020: *M* = 86.77, *SD* = 4.54) with three points of measurement from May to June 2020 (t1), March to May 2021 (t2) and November to January 2022 (t3). Besides sociodemographic variables, worries about the Sars-Cov-2 virus, living situation, perceived social support (ESSI), resilience (BRS), anxiety and depressive symptoms (BSI-18) were assessed. We calculated multilevel mixed-effects generalized linear models with a negative binominal distribution to model anxiety and depressive symptoms over time.

**Results:**

While there is an increase in depressive and anxiety symptoms in the investigated oldest-old individuals in Germany from 2020 to 2021, there is no further increase in symptomatology from 2021 to 2022. Participants of older age reported higher levels of anxiety symptoms. Higher perceived social support was associated with both less depressive and less anxiety symptoms, while resilience was associated with less depressive symptoms only. More worries about the Sars-Cov-2 virus were associated with higher anxiety levels.

**Conclusion:**

Overall, the oldest-old population appeared to show rather stable mental health after a slight increase in symptomatology within the first year of the pandemic. Social support is an important factor to target in mental health prevention programs for oldest-old individuals in times of future crises such as a pandemic.

## 1 Introduction

The COVID-19 (Coronavirus Disease 2019) pandemic and associated governmental measures, like lockdowns and social distancing, impacted everyday lives all over the world tremendously, and serious mental health consequences were expected (1). People of old and oldest-old age were identified as a high-risk group regarding a severe course of Sars-Cov-2 virus disease and a heightened mortality risk (2, 3). Although this group had to deal with a higher threat to their health, findings on their mental health are scarce and show heterogeneity. While some studies postulated a rather stable level of mental health factors, such as depressive and anxiety symptoms, in old age leading to an assumption of mental resistance (4–6), other studies point out certain mental vulnerabilities, e.g. in terms of an increase in mental distress symptomatology of the old age population (7–11). First evidence predominantly exists for the first year of the pandemic, and mostly people aged 80 years and older are either not included in the study samples or make up a rather small proportion of the sample so that results cannot shed light on the mental health of oldest-old individuals during the pandemic. We do have first evidence suggesting an increase of anxiety symptoms in a sample with a mean age of 63.13 (*SD* = 6.01) within the beginning of the pandemic (9). A cross-sectional study by Parlapani et al. showed a high percentage of moderate to severe anxiety (84.5%) and depressive symptoms (81.6%) in older individuals with a mean age of 69.85 (*SD* = 5.26) during the beginning of the pandemic (10). In regards to depressive symptoms (2, 3), Briggs and colleagues (7) found a significant increase in depressive symptomatology, with an age older than 70 years and/or living alone being associated with more depressive symptoms. Wettstein et al. (11) analyzed data of older adults in Germany (mean age in 2020: 69.9 years, *SD* = 10.4) and reported an increase in depressive symptoms, especially for those who showed higher concerns about the pandemic. Recently published findings of our study group showed an increase in depressive and anxiety symptoms from March 2020 to May 2021 (8).

Whereas these studies give reason to assume a mental health burden in old and oldest-old age during the coronavirus pandemic, research also gives points of reference for older age being associated with less psychological distress (12–14). When taking a look at a more strength-oriented as opposed to the deficit-oriented views, old age also comes along with more life experience and may result in a more differentiated repertoire of coping skills or even more placid and calm view of crises (5, 15, 16).

While social isolation illustrates a severe risk factor for people’s mental health, especially in old age (17, 18), we aim to investigate an indicator of social inclusion as a potential protective factor for mental health in old and oldest-old age. According to past studies’ findings during the pandemic, social support is associated with less psychological distress, e.g. in terms of depressive and/or anxiety symptoms, in younger (19, 20) and older adults (21–23).

In the studies mentioned above investigating mental health and potential protective factors, either the age group of individuals aged over 80 years old is not included or it makes up a rather small proportion in the investigated samples, or the investigated time frame only includes data up to May 2021. To be able to fill the existing gap in scientific literature, in our study, we aim to investigate how depressive and anxiety symptoms develop in old and oldest-old individuals during the pandemic over a course from May 2020 to January 2022 in Germany. Additionally, our aim is to shed light on the relationship between perceived social support and perceived resilience as potential protective factors with depressive and anxiety symptoms. Findings could help us to inform public health decisions and interventions on how to prevent adverse mental health event in crises like the pandemic that are tailored to the specific needs of the old and oldest old population. Given that this population will continue to grow in the next decades and will potentially make up one fifth of the general population (24) it should be of great public health interest to prevent mental distress and facilitate mental well-being in this age group.

Taken together, we aim to answer the following research questions:

- Are there significant increases in depressive and anxiety symptoms in the sample of oldest-old individuals over the course of the pandemic from March 2020 to January 2022?
- How are psychosocial factors such as worries about the pandemic, living alone, social support and resilience associated with depressive and anxiety symptoms during the pandemic?

## 2 Methods

### 2.1 Study design, procedure and participants

**Figure 1:**
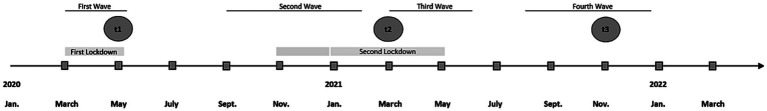
Measurement points over the course of the COVID-9 pandemic and times of lockdowns. Classification of COVID-19 waves according to the Robert Koch Institute, Germany (25).

In the current study, we analyze longitudinal data that was collected via paper-pencil based assessments that were sent to the participants’ homes. Times of measurement were closely after the first wave of the COVID-19 pandemic from May to June 2020 (t1), during the third COVID-19 wave from March to May 2021 (t2) and during the fourth COVID-19 wave from November 2021 to January 2022 [t3; classification of COVID-19 pandemic waves in Germany according to the Robert Koch Institute; also see Figure 1 (25)]. Initially, 378 people aged 78 years and older were contacted. The survey pool, where the 378 people were taken from, consisted of former study participants that took part in recent studies conducted by the institute and had agreed to being contacted for future studies. Out of the contacted people, 197 (52.12%) returned a filled out questionnaire for t1. For t2, 156 participants (79.19%) took part in the assessment, and for t3, 135 participants (86.54%) filled out the questionnaire. The non-responder analysis showed there were no differences in age (*t* = 0.825, *p* = 0.410) nor gender (*x*^2^ = 0.702, *p* = 0.402) between responders and non-responders at t1. Non-responder analysis at t2 showed that there was a significant difference in age between responders and non-responders (*t* = 3.928, *p* < 0.001) with non-responders being older compared to responders and no statistical significant difference in gender (*x*^2^ = 3.797, *p* = 0.051) with a tendency toward more female non-responders compared to male non-responders. At t3, the non-responder analysis showed no significant difference in gender between responders and non-responders (*x*^2^ = 0.160, *p* = 0.689), but a significant difference in age (*t* = 3,009, *p* = 0.003) with non-responders being older compared to responders.

In the current study, we analyzed data of participants who took part in all three assessments (*n* = 135). All study participants signed a written informed consent statement. The study was approved by the Ethics Committee of the Medical Faculty of the University of Leipzig (ethic approval number: 206–20-ek). Further information on the study design and data collection can also be retrieved from recent publications (8, 26).

### 2.2 Measures

Depressive and anxiety symptoms. Depressive symptoms and anxiety was measured with the subscales “depression” and “anxiety” of the 18-Item-Version of the Brief Symptom Inventory (27, 28). The two subscales consist of six items each that are rated on a five-point-Likert Scale (0 to 4, “not at all” to “very much”). Sum scores were calculated. The psychometric properties of the BSI subscales for anxiety and depressive symptoms can be described as good with Cronbach’s alpha values of α = 0.87 for depressive and α = 0.84 for anxiety symptoms in a representative German sample (28, 29). In our study, the Cronbach’s alpha was α = 0.53, α = 0.73 and α = 0.78 for the depressive symptom scale and α = 0.53, α = 0.66 and α = 0.69 for the anxiety symptoms scale.

Perceived social support. The extent of perceived social support was measured using the ENRICHD Social support Inventory (30). Five items were rated on a five-point Likert Scale (1 to 5, “never” to “always”). Sum scores were calculated. The scale has proven its good psychometric properties (30). In our study, the Cronbach’s alpha ranged between α = 0.83 and α = 0.86.

Perceived resilience. The Brief Resilience Scale (31) assessed the extent of perceived resilience, which is the perceived ability to bounce back from stressful events. Six items are rated on a five-point Likert Scale (1 to 5, “fully agree” to “fully disagree”). Mean scores were calculated. In line with Chmitorz et al. (32), we adjusted for a method factor. Evidence for good reliability and validity of the scale was provided by Chmitorz et al. (32). In our study, the Cronbach’s alpha was between α = 0.66 and α = 0.75.

COVID-19-specific worries. The extent of worries regarding the Coronavirus was assessed with a single item (“I am worried because of the Coronavirus”) that could be rated on a five-point Likert Scale (0–4, “fully agree” to “fully disagree”).

Living Alone. The living situation was assessed with a single item (“Do you live alone or with someone in a common household?,” 1 = living alone, 0 = living with someone).

Education. Educational levels were categorized into low, medium and high educational levels according to the Comparative Analysis of Social Mobility in Industrial Nations [CASMIN; (33)].

Marital status. The marital status of the participants was assessed through a single item question (“What is your marital status at this moment?”) and answers were categorized in married, divorced/single and widowed.

### 2.3 Data analysis

Firstly, descriptive statistics of all sociodemographic and psychosocial factors were analyzed. Results are shown as means and standard deviations or frequencies and percentages, as appropriate. Secondly, the distributions of the outcome variables for the number of depression and anxiety symptoms were examined and identified as negative binominal. We then selected two multilevel mixed-effects generalized linear models with negative binomial distributions and random intercepts to analyze associations of (1) time of measurement with depressive and anxiety symptoms, and (2) time of measurement, age (metric), sex (male in reference to female), education (middle and high in reference to low), marital status (single/divorced and widowed in reference to married), worries about the Sars-CoV-2 virus (metric), living alone (living alone in reference to living with someone), social support (metric) and resilience (metric) with depressive and anxiety symptoms, each at the respective wave, except those that are time-stable such as sex and education. We reported Incident Rate Ratios (*IRR*), 95%-Confident Intervals (95%-*CI*), *z*-values and *value of p*s considering *value of p*s below 0*.05* as significant. We performed *x*^2^-omnibus tests for all categorical variables. Robust standard errors were used for all models. The statistical analysis was conducted with Stata SE 16.0 and SPSS 27.0 (34, 35).

## 3 Results

### 3.1 Sample characteristics

The sociodemographic and psychosocial characteristics of the analysis sample divided by time of measurement can be retrieved from Table 1. The mean age at measurement t1 was 86.77 (*SD* = 4.54) with an age range of 78 to 97 years. 57% were female and the educational level was distributed quite evenly, with slightly more participants having a high educational level (38.6%). Most participants were either married (44.4% at t1) or widowed (45.9% at t1). At time point t1 no one was infected with the SarS-Cov-2 virus, but one person (1.35%) reported a family member that was infected and in quarantine. By spring 2021 (t2) one person (1.35%) reported having been infected with the virus but none reported being in quarantine at the time of measurement. There was also one person reporting a member of the household was infected (1.35%) and one household member was in quarantine (1.35) at the time of measurement. In the winter of 2021/2022 one person reported being infected with the virus and being in quarantine (1.31%) and four participants did not answer the questions regarding infections or quarantine. One participant also reported that a member of the household was infected with the virus and in quarantine (1.31%).
Sex-specific analysis (see Appendix) showed that women more often lived alone compared to men at all three points of measurement (t1: *x*^2^ = 17.51, *p* < 0.001; t2: *x*^2^ = 13.86, *p* < 0.001; t3: *x*^2^ = 13.71, *p* < 0.001). Men reported slightly more social support compared to women at t1 (*U* = 2580.50, *p* = 0.022) and t2 (*U* = 2573.50, *p* = 0.040), but there was no significant difference in perceived social support at t3. Furthermore, men reported slightly higher perceived worries about the virus compared to the female participants at the first measurement (t1; *U* = 2513.00, *p* = 0.050), but there were no differences at the other two points of measurement. Men reported slightly higher resilience compared to women at t1 (*U* = 2623.50, *p* = 0.037). There were no differences at t2 or t3. Moreover, there were no sex-differences in reported depressive or anxiety symptoms at the three measurements except from higher reported depressive symptoms of women compared to men at measurement t2 (*U* = 1592.00, *p* = 0.016). More detailed information on sex differences can be retrieved from the Appendix Table.

**Table 1:** Sociodemographic and psychosocial characteristics of the analyzed sample (*n* = 135).

	t1 summer 2020	t2 spring 2021	t3 winter 2021/2022
Sociodemographic factors			
Age; *M (SD)*	86.77 (4.54)	87.60 (4.55)	87.81 (4.51)
Sex; *n (%)*			
Female	77 (57.0)		
Male	58 (43.0)		
Marital status; *n (%)*			
Married	60 (44.4)	60 (44.4)	58 (43.0)
Single/divorced	13 (9.6)	12 (8.9)	14 (10.37)
Widowed	62 (45.9)	63 (46.7)	63 (46.7)
Education (CASMIN); *n (%)*			
Low	47 (35.6)		
Medium	34 (25.8)		
High	51 (38.6)		
Psychosocial factors			
Worries about the virus; *M (SD)*	2.77 (1.14)	2.90 (1.14)	3.18 (1.05)
Living alone; *n (%)*	67 (50.00)	65 (48.51)	69 (51.11)
Social support^a^; *M (SD)*	22.17 (3.51)	22.50 (3.30)	21.99 (3.51)
Resilience^b^; *M (SD)*	3.40 (0.70)	3.35 (0.64)	3.43 (0.64)
Depressive symptoms^c^; *M (SD)*	0.41 (1.13)	2.13 (2.77)	2.19 (3.01)
Anxiety symptoms^c^; *M (SD)*	0.29 (0.77)	2.24 (2.41)	2.35 (2.72)

M mean; SD standard deviation; ^a^Enriched Social Support Inventory ESSI; ^b^Brief Resilience Scale BRS; ^c^Brief Symptom Inventory BSI-18 subscale depressive and anxiety symptoms; missing values. Education: *n* = 3 (2.2%), depressive symptoms: t1, t2: *n* = 5 (3.70%), t3: *n* = 4 (2.96%); anxiety symptoms: t1: *n* = 4 (2.96%), t2: *n* = 8 (5.93%), t3: *n* = 5 (3.70%); worries: t1: *n* = 4 (2.96%), t2: *n* = 3 (2.22%), t3: *n* = 6 (4.44%); social support: t1: *n* = 4 (2.96%), t2: *n* = 3 (2.22%), t3: *n* = 6 (4.44%); resilience: t1: *n* = 2 (1.48%), t2: *n* = 5 (3.70%), t3: *n* = 8 (5.93%).

### 3.2 Depressive and anxiety symptom development over time

**Figure 2:**
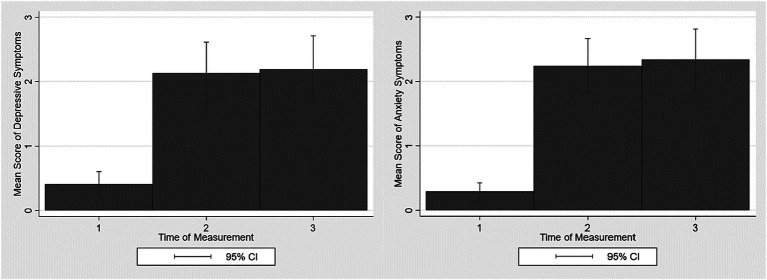
Increases in depressive and anxiety symptoms in the analyzed sample of old age during the Covid-19 pandemic (*n* = 135). 95% CI = 95% Confidence intervals.

There is a graphic illustration of increases in depressive and anxiety symptoms in Figure 2. Graphically and descriptively, we see an increase in depressive and anxiety symptoms from the first time of measurement in summer 2020 (t1) to the second time of measurement in spring 2021 (t2) and a further very slight increase at the third time of measurement in winter 2021/2022 (t3; also see Table 1).

The multilevel mixed-effects generalized linear model for depressive symptoms is shown in Table 2 and shows a significant association of time of measurement and depressive symptoms with higher scores of depressive symptoms at t2 compared to t1 (*IRR* = 5.422, *z* = 8.16, *p* < 0.001) and higher scores of depressive symptoms at t3 compared to t1 (*IRR* = 5.591, *z* = 7.72, *p* < 0.001). When switching the reference category to t2, there was no significant difference regarding depressive symptoms from t3 in reference to t2 (*IRR* = 1.031, *z* = 0.34, *p* = 0.733) with a small but not significant trend toward a marginal increase.

**Table 2:** Multilevel mixed-effects generalized linear model for the association of the time of measurement and depressive and anxiety symptoms in an old age sample during the COVID-19 pandemic in Germany.

	Depressive symptoms (*n* = 134)				Anxiety symptoms (*n* = 134)			
	*IRR*	*95%-CI*	*z*	*p*	*IRR*	*95%-CI*	*z*	*p*
Time of Measurement (ref. t1)		*x*^2^(2) = 66.97, *p* < 0.001				*x*^2^(2) = 103.33, *p* < 0.001		
t2	5.422	3.61–8.14	8.16	**<0.001**	7.931	5.21–12.07	9.66	**<0.001**
t3	5.591	3.61–8.65	7.72	**<0.001**	8.073	5.38–12.11	10.10	**<0.001**

The multilevel mixed-effects generalized linear model for anxiety symptoms is also shown in Table 2. There is a significant association of time of measurement and anxiety symptoms with higher scores of anxiety symptoms at t2 compared to t1 (*IRR* = 7.931, *z* = 9.66, *p* < 0.001) and higher scores of anxiety symptoms at t3 compared to t1 (*IRR* = 8.073, *z* = 10.10, *p* < 0.001). When switching the reference category to t2, there was no significant difference regarding anxiety symptoms from t3 in reference to t2 with a marginal but not significant trend toward a slight increase in anxiety symptoms (*IRR* = 1.018, *z* = 0.20, *p* = 0.844).

### 3.3 Psychosocial characteristics and depressive symptoms

Results of the multilevel mixed-effects generalized linear model with the outcome of depressive symptoms are shown in Table 3. When adjusting for psychosocial variables worries about the virus, living alone, social support and resilience, we still see the significant effect of time of measurement. There is no significant association of more worries (*IRR* = 1.103, *z* = 1.27, *p* = 0.204) nor the living situation of living alone (*IRR* = 1.632, *z* = 1.37, *p* = 1.72) with depressive symptoms. Higher perceived social support is associated with less depressive symptoms (*IRR* = 0.886, *z* = −4.40, *p* < 0.001). Higher perceived resilience is associated with less depressive symptoms (*IRR* = 0.511, *z* = −2.84, *p* = 0.005).

**Table 3:** Multilevel mixed-effects generalized linear model for the association of sociodemographic and psychosocial factors and depressive and anxiety symptoms in an old age sample during the COVID-19 pandemic in Germany.

	Depressive symptoms (*n* = 130)				Anxiety symptoms (*n* = 131)			
	*IRR*	*95%-CI*	*z*	*p*	*IRR*	*95%-CI*	*z*	*p*
Time of Measurement (ref. t1)		*x*^2^(2) = 79.49, *p* < 0.001				*x*^2^(2) = 70.48, *p* < 0.001		
t2	5.767	3.937–8.535	8.90	**<0.001**	8.782	5.169–14.235	8.32	**<0.001**
t3	5.738	3.643–9.040	7.54	**<0.001**	8.094	4.890–13.397	8.13	**<0.001**
**Sociodemographic factors**								
Age	0.994	0.941–1.050	−0.22	0.826	1.048	1.011–1.086	2.56	**0.010**
**Sex (ref. female)**								
Male	0.925	0.551–1.553	−0.29	0.768	0.785	0.521–1.181	−1.16	0.245
Education (ref. low)		*x*^2^(2) = 0.54, *p* = 0.765				*x*^2^(2) = 4.90, *p* = 0.087		
Middle	1.207	0.700–2.081	0.68	0.498	1.502	0.982–1.296	1.88	0.060
High	1.038	0.579–1.861	0.13	0.900	1.023	0.654–1.601	0.10	0.920
Marital Status (ref. married)		*x*^2^(2) = 0.01, *p* = 0.997				*x*^2^(2) = 0.64, *p* = 0.728		
Single/divorced	0.971	0.396–2.385	−0.06	0.950	0.780	0.354–1.178	−0.62	0.537
Widowed	0.993	0.478–2.062	−0.02	0.984	0.742	0.356–1.547	−0.80	0.425
**Psychosocial factors**								
Worries about the Sars-Cov-2 virus	1.103	0.948–1.284	1.27	0.204	1.282	1.109–1.482	3.36	**0.001**
Living Alone (ref. with someone)	1.632	0.808–3.294	1.37	0.172	1.594	0.803–3.164	1.33	0.183
Social Support	0.886	0.839–0.935	−4.40	**<0.001**	0.953	0.913–0.996	−2.15	**0.031**
Resilience	0.511	0.322–0.812	−2.84	**0.005**	1.151	0.839–1.581	0.87	0.385
Method factor	1.041	0.798–1.358	0.30	0.766	0.803	0.654–0.986	−0.2.09	0.036

Omnibus tests were performed for categorical variables.

### 3.4 Psychosocial characteristics and anxiety symptoms

Results of the multilevel mixed-effects generalized linear model with the outcome of anxiety symptoms can be retrieved from Table 3 as well. The effect of time of measurement remains when adjusting for psychosocial variables. Increased age is more likely associated with higher anxiety (*IRR* = 1.048, *z* = 2.56, *p* = 0.010). A higher extent of worries about the Sars-CoV-2 virus is associated with higher anxiety levels (*IRR* = 1.282, *z* = 3.36, *p* = 0.001) and higher perceived social support is associated with less anxiety (*IRR* = 0.953, *z* = −2.15, *p* = 0.031). There is no significant association of living alone with anxiety symptoms (*IRR* = 1.282, *z* = 1.33, *p* = 0.183), nor of resilience with anxiety symptoms (*IRR* = 1.151, *z* = 0.87, *p* = 0.385).

## 4 Discussion

This study aimed to explore the development of depressive and anxiety symptomatology in individuals of the oldest-old population in Germany during the COVID-19 pandemic with measurement points from 2020 to 2022. Additionally, sociodemographic and psychosocial factors and their association with the extent of depressive and anxiety symptoms were investigated.

Results show an increase in depressive symptomatology from 2020 to 2021 and a non-significant change in symptom levels from 2021 to 2022. This result exists with and without including sociodemographic and psychosocial factors in the model. The increase in depressive symptomatology is in line with results reported by Briggs and colleagues (7), with the exception that in our study living alone was not associated with more depressive symptoms. This may be due to the fact that the sample analyzed in our study was particularly well socially integrated and those who lived alone had social contacts with people beyond their household, so that the factor of the living situation might not have been as relevant as in the study of Briggs et al. (7). Wettstein et al. (11) investigated a German sample of older adults and results show this increase in depressive symptoms levels as well. They also report an association between concerns about the pandemic and depressive symptoms. In our study, the related construct of worries about the virus was not significantly associated with higher depressive symptomatology. However, it was associated with more anxiety symptoms. This is in line with findings of a previous study during the COVID-19 pandemic (21). A possible explanation could be the different worry content. Diefenbach et al. distinguish anxious from depressive worry content (36). For our results, this could mean that the nature of worries was of a more anxious kind, meaning the worries’ content referred to, e.g., a lack of control about the course of the pandemic and associated circumstances like lockdowns. Taking in account the overall lower mental distress levels in terms of depressive and anxiety symptoms this could be interpreted as a reaction to the objective threat by the disease and the uncertainty of the progression of the pandemic.

In 2021 (second measurement, t2) women reported slightly higher depressive symptoms compared to men, in winter 2021/2022 (third measurement, t3) this significant difference vanishes. A possible explanation for this brief difference might be the traditional gender roles, where the men might have taken over activities like grocery shopping and pharmacy visits for their spouse to take care of them and avoid having them exposed to the virus. This might potentially have been associated with more social engagement for the men, resulting in slightly higher levels of perceived social support and slightly lower depressive symptom levels at the second measurement in 2021. Later there might have been more habituation to the pandemic situation and both spouses engaged in social activities equally again. Since our sample was rather small, future studies with larger samples should investigate specific gender differences in the reaction and adaption to pandemic crises as well as predictive factors of mental health factors in the oldest-old population.

The level of anxiety symptoms increased from 2020 to 2021. Parallel to the course of development of depressive symptoms, we then see that the anxiety levels stay relatively stable from 2021 to 2022. The aggravation of anxiety symptomatology from 2020 to 2021 is in line with the reported increase of anxiety symptoms by Parlapani et al. (10) and Gosselin et al. (9).

Mauz et al. investigated depressive and anxiety symptoms in the general adult population during the pandemic (37). They describe an increase in depressive and anxiety symptomatology during the second wave of the pandemic in October 2020, constant levels throughout 2021 and a second increase from late 2021 to spring 2022 in the observed adult general population. While the author describe a continuous increase in the subgroup of older adults (64+ years), we see an increase in spring 2021 (t2) followed by a non-significant change of depressive and anxiety symptomatology, indicating a stabilization of mental distress in the old and oldest-old individuals. This leads to the assumption that the mental health development of old and oldest-old individuals shows heterogeneity in different subgroups who may differ in specific characteristics like for example preexisting medical, psychiatric or cognitive conditions. There is an urgent need to investigate this heterogeneous group to identify vulnerable groups within the old and oldest-old population and to enable decision makers to design public health interventions accordingly.

It is important to mention that despite increased symptomatology levels up to the measurement in 2021 (t2), they can still be interpreted as quite low and seem to stay relatively stable after the slight increase. Röhr et al. (5) investigated a sample of *n* = 1,005 individuals aged 65 years and older during the beginning of the pandemic and reported mean depressive symptom scores of *M* = 1.4 (*SD* = 2.0), vs. *M* = 2.1 (*SD* = 2.8) at the t2 measurement in our study, and mean anxiety scores of *M* = 1.6 (*SD* = 2.0), vs. *M* = 2.2 (*SD* = 2.4) at the t2 measurement in our study. With depressive and anxiety symptom mean scores of 2.1 (*SD* = 2.8) and 2.2 (*SD* = 2.4) in our investigated sample at the second point of measurement and a possible range of 0 to 24, the manifestation of depressive and anxiety symptoms can be described as low. The depressive symptom score of 96.4% of the participants was located in the lower 25% (score ≤ 6) of the scale. For anxiety symptoms, 92.0% of the participants showed scores located in the lower 25% (score ≤ 6) of the possible sum score range at the second point of measurement. At the third point of measurement the mean score of depressive and anxiety symptoms was 2.19 (SD = 3.01) and 2.35 (SD = 2.72), and can still be considered as low with 90.8% (depressive symptoms) and 90.0% (anxiety symptoms) of the participants scoring in the lower 25% of the possible sum score range. Oldest-old individuals seem to be of generally good mental health despite the pandemic and only show a slight increase in psychological distress in terms of depressive and anxiety symptoms within the first year of the pandemic, followed by a relatively stable level of mental health burden. The further development should be investigated and monitored in future studies.

Contrary to studies reporting an association of increased age with lower mental health burden (12–14), in our investigation we see that increased age is associated with higher anxiety levels. However, taken together with the overall lower general anxiety levels, this pattern of associations might hint to what is a natural reaction of the oldest-old group to the objectively higher risk of a severe course of the Coronavirus-disease and the higher mortality risk.

This is also in line with the paradox of aging, with older individuals reporting generally high mental well-being despite physical and cognitive limitations that come with age (38); in our study during the pandemic this was despite the high risk for a worse course of disease.

High perceived resilience was associated with lower depressive symptom levels but not with anxiety symptom levels. In line with Röhr et al. (5) and Gerhards et al. (21), this means that perceived resilience can be a protective factor against increases in depressive symptoms. Contrary to Röhr et al. (5) and Gerhards et al. (21), we could not find this for perceived resilience and anxiety symptoms. This might be due to the fact, that this effect only appears when experiencing higher levels of anxiety and not when levels are quite low and part of a normal reaction to threat. Further studies are needed to clarify this finding.

Social support does seem to be a protective factor against anxiety and depressive symptoms in our analyzed study sample of older individuals, since higher social support is associated with lower levels of depressive and anxiety symptomatology. The finding is concurrent with previous research (19–23). Where van Tilburg et al. (6) reported increasing levels of loneliness, our investigated sample showed high levels of social support while reporting generally low levels of mental distress in form of anxiety and depressive symptoms. A fitting explanatory theory could be the socioemotional selectivity theory by Carstensen and colleagues (39) that postulated a shift of focus from gaining knowledge to emotional regulation of related goals, which is accompanied by a shift of focus from multiple contacts to just a few very close ones. For the interpretation of the current study, such findings would mean that the oldest-old individuals might have been content with having only very few social contacts with their loved ones (e.g., partner or family member) which could presumably also be maintained during lockdowns, resulting in a high perceived social support. This may have been followed by relatively low general mental distress in form of anxiety and depressive symptoms.

The increase in the beginning could be interpreted as a normal psychological reaction to an objective threat. Future studies should still analyze the further development of mental distress in the oldest old population to rule out an aggravation of symptomatology and to identify vulnerable groups in larger samples. While our study investigates an underrepresented study sample during the COVID-19 pandemic and uses longitudinal data, there are some limitations that need to be mentioned as well. The study sample is of a rather small size due to high dropout rates in the oldest-old group, either because of medical conditions that made the participant unable to fill out the questionnaires or death due to old age. We also were not able to control for (pre-) existing medical or psychiatric conditions or cognitive status. Since the participants were able to fill out and file the questionnaire, it is to be assumed that the cognitive status was sufficient. Further studies may investigate the development of mental health factors up to the late phase of the COVID-19 pandemic in late 2022 and the beginning of 2023 to give information on the progression of anxiety and depressive symptomatology and may additionally aim at identifying possible gender differences in the prediction of depressive and anxiety symptoms in larger samples.

## 5 Conclusion

The study contributes to a better understanding of the psychological health and psychological adaption of oldest-old individuals in Germany during the COVID-19 pandemic who are largely underrepresented in the scientific research field of public mental health during the pandemic. It highlights the importance of social support for mental health during crises and elicits valuable starting points for further research and prevention programs to support the oldest-old individuals’ mental health. Overall, our study findings show, that after a first increase in depressive and anxiety symptoms from 2020 to 2021, we see a stagnation of symptoms, indicating a rather good and stable mental health of the oldest-old individuals during the pandemic from midst of 2021 to the beginning of 2022. Findings show that being and feeling socially integrated is an essential influencing factor for the oldest-old individuals’ mental health and public health decisions and interventions should takes this into account by for example promoting activities to facilitate the feeling of social support. Moreover, it may be advantageous for promoting mental health and averting aggravation of depressive symptoms to highlight resources, abilities and coping skills that the oldest-old individuals have gained over the years to strengthen the feeling of being resilient and self-efficient.

## Data availability statement

The raw data supporting the conclusions of this article will be made available by the authors, without undue reservation.

## Ethics statement

The studies involving humans were approved by Ethics Committee of the Medical Faculty of the University of Leipzig. The studies were conducted in accordance with the local legislation and institutional requirements. The participants provided their written informed consent to participate in this study.

## Author contributions

SRH and ML Conceptualizing and designing the study. ML Data collection and supervision of data collection. SKG Analysis, Interpretation of results and writing the manuscript. AP Methodical and statistical advice. ML, AP, and SRH revising the manuscript. All authors contributed to the article and approved the submitted version.
