# Supplementary material for: Depressive and anxiety symptoms during the COVID-19 pandemic in the oldest-old population and the role of psychosocial factors: a multivariate and multi-wave analysis
Source: Front Public Health. 2023 Dec 15;11:1229496. doi: 10.3389/fpubh.2023.1229496 (PMC10773748; doi:10.3389/fpubh.2023.1229496)
Supplement: Supplementary file 1 [file Table_1.DOCX]

**Appendix A.** Gender Differences in the analyzed sample (n=135).

|  | **t1**  Summer 2020 | | | | | **t2**  Spring 2021 | | | | | **t3**  Winter 2021/2022 | | | |
| --- | --- | --- | --- | --- | --- | --- | --- | --- | --- | --- | --- | --- | --- | --- |
|  | female | male | test statistic | group difference (p-value) | female | | male | test statistic | group difference (p-value) | female | | male | test statistic | group difference (p-value) |
| Age; M(SD) | 86.16 (4.46) | 87.58 (4.55) | 2645.50 | .067 | 87.03 (4.46) | | 88.36 (4.59) | 2619.00 | .086 | 87.17 (4.45) | | 88.66 (4.50) | 2672.50 | .050 |
| Marital status; n (%) |  |  | 15.47 | <.001 |  | |  | 15.58 | <.001 |  | |  | 15.15 | <.001 |
| Married | 23 (29.9) | 37 (63.8) |  |  | 23 (29.9) | | 37 (63.8) |  |  | 22 (28.6) | | 36 (62.1) |  |  |
| Single/divorced | 9 (11.7) | 4 (6.9) |  |  | 8 (10.4) | | 4 (6.9) |  |  | 10 (13.0) | | 4 (6.9) |  |  |
| Widowed | 45 (58.4) | 17 (29.3) |  |  | 46 (59.7) | | 17 (29.3) |  |  | 45 (58.4) | | 18 (31.0) |  |  |
| Worries about the virus; M (SD) | 2.59 (1.18) | 3.00 (1.05) | 2513.00 | .050 | 2.80 (1.20) | | 3.04 (1.04) | 2335.50 | .314 | 3.10 (1.06) | | 3.29 (1.04) | 2271.50 | .234 |
| Living alone; n (%) | 48 (62.34) | 17  (29.82) | 17.51 | <.001 | 48 (62.3) | | 17 (29.3) | 13.86 | <.001 | 50 (64.9) | | 19 (32.8) | 13.71 | <.001 |
| Social Support; M (SD) | 21.37 (4.21) | 23.23 (2.05) | 2580.50 | .022 | 22.04 (3.43) | | 23.09 (3.03) | 2573.50 | .040 | 21.58 (3.60) | | 22.55 (3.33) | 2387.50 | .087 |
| Resilience; M (SD) | 3.29 (.71) | 3.55 (.67) | 2623.50 | .037 | 3.33 (.63) | | 3.41 (.60) | 2366.50 | .134 | 3.34 (.63) | | 3.55 (.64) | 2357.00 | .059 |
| Depressive Symptoms; M (SD) | .533 (1.28) | .236  (.88) | 1789.00 | .065 | 2.65 (3.15) | | 1.48 (2.07) | 1592.00 | .016 | 2.32 (2.82) | | 2.02 (3.26) | 1866.00 | .260 |
| Anxiety Symptoms; M (SD) | .378  (.89) | .175  (.57) | 1924.00 | .170 | 2.53 (2.55) | | 1.87 (2.18) | 1681.50 | .137 | 2.57 (2.93) | | 2.05 (2.40) | 1898.00 | .404 |
| *Notes*. Group differences were calculated with chi-square tests for marital status and living alone and with Mann-Whitney-U tests for age, worries about the virus, social support, resilience, depressive and anxiety symptoms. | | | | | | | | | | | | | | |
